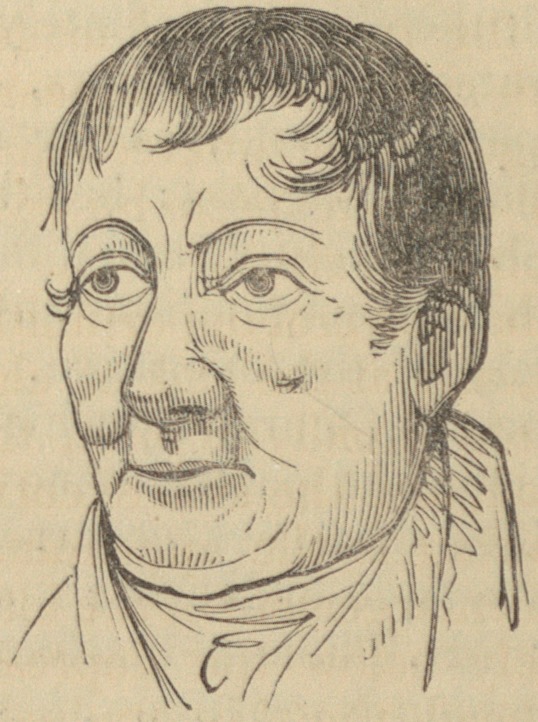# On the Removal of Morbid Enlargements of the Integuments of the Nose

**Published:** 1834-01-01

**Authors:** John Dalrymple

**Affiliations:** Assistant Surgeon to the Ophthalmic Infirmary.


*On the Removal of Morbid Enlargements of the Integuments of the Nose.* By John Dalrymple, Esq., Assistant Surgeon to the Ophthalmic Infirmary.

Morbid enlargements of the integuments of the nose are not of infrequent occurrence in persons somewhat past the middle period of life; while at the same time it appears that the female is exempt from this unsightly disease. The growth of skin often proceeds to a very considerable extent, and not only produces great personal deformity, but also distressing physical inconvenience. The appearance of a person thus affected is familiar to every one, and he is generally believed, though often most unjustly, to have earned this mark as characteristic of the intemperance of his habits. It is but seldom, however, that the patient feels it necessary, except in the most aggravated cases, to have recourse to medical advice; and but rarely indeed does it occur to his mind, that an operation would relieve him from an inconvenience, that has been familiarised by the slowness of its growth, not only to himself, but to his relations and acquaintance.

Instances of *decorative surgery* are met with in the Taliacotian operation; although, in the most successful cases, the appearance of the restored member is but little flattering to the vanity of the patient. Another variety of this operation has been fully successful in the hands of Mr. Liston, of Edinburgh, where a permanent fissure of the alæ of the nose and the loss of the median column was repaired by his skill. Mr. Hey, in his excellent Practical Observations on Surgery, has described an operation for the removal of a tumour of the nose of the kind which I am discussing. He has cited the authority of the French surgeons,* as regards the safety and practicability of such a step, although he differs from them, and very justly, as to the malignant character of these morbid growths.

Although I believe this operation to be unattended with danger to life, yet, as it must be admitted to be exceedingly painful, and occasionally not a little tedious in the performance, the surgeon would hardly be justified in recommending it to the patient, unless the result were to be something more than the removal of a mere personal deformity. Several cases, however, occur in which great inconvenience, and even suffering, are produced by the excessive growth of the nasal integuments; and, if we place out of view the exclusion from general society which such a malady may entail upon the sufferer, yet the difficulty of breathing, the impediment to the speech, and the occasional ulceration of the surface of the tumour, sometimes render the removal of the diseased mass absolutely necessary.

This disease cannot be called simple hypertrophy of the skin, since this tissue has lost its pliancy and natural colour; but rather approaches to a state of elephantiasis, in which the cellularity is partially destroyed, and a fibro-cellular structure substituted. The mass presents externally a nodulated surface of a purple or deep red colour, traversed by numerous minute and tortuous vessels. The larger separated portions are frequently divided from each other by deep fissures, occupying in many cases the convexities of the alæ and extremity of the nose. Where the disease has been of long standing, the altered state of skin advances as high as the function of the frontal with the nasal integuments, seldom encroaching much on the palpebral furrow laterally, but accompanied, in the majority of instances, by a wattled state of the skin of the cheek, corresponding in colour and general appearance with the tumour of the nose. The sebaceous follicles are greatly enlarged, and their secretion is not only increased in quantity, but, unless extreme cleanliness is attended to, it is offensive in smell, and excoriates the surrounding skin.

* Vide Mem. de l'Academie de Chirurgie, tom. iii., p. 511; and the New Progress of Surgery in France, by J. Delonnes, M.D., translated by T. Chavernac, surgeon.

As the disease proceeds, the tumour becomes pendulous, hanging down in one or more masses to the level of the lips, and even below them, so that the nose requires to be suspended in the act of taking the food, or in drinking from a glass. At this time the patient is much inconvenienced by the pressure upon the nostrils, and he ceases to breathe by these apertures. The speech becomes troubled, while at night the respiration is noisy, and almost stertorous. If the patient be far advanced in life, or the vital powers begin to fail, the skin immediately around the sebaceous openings ulcerates. These ulcers, or rather excoriations, are however superficial, and, as far as my observations lead me, never become malignant; but they are sufficiently troublesome from their irritability, and difficult to heal. The following cases, in which amputation was resorted to, will demonstrate the safety of the operation, and the rapidity of the subsequent process of cure, by which the patients were relieved from a serious and painful annoyance.

The first case occurred in the practice of my father, Mr. Dalrymple of Norwich, and I assisted at the operation.

"Mr. Thomas Platfoot is fifty-five years of age; his occupation is sedentary, his constitution sound, his habits of life are temperate.

He has a considerable enlargement of his nose, which began, without any assignable cause, about eleven years ago. The swelling is exceedingly unsightly; it falls down over his mouth, below the level of his lower lip; it troubles his respiration especially during sleep, and is extremely inconvenient to him when eating.

As in a similar case described by Mr. Hey, the disease appears to consist of simple enlargement of the common integuments of the parts. The mass is of an irregular, nodulated form, of the deepest red colour, and very vascular. The orifices of the sebaceous follicles are very numerous and large, and give to the eye the appearance of common cuticle viewed through a strong magnifier.

The removal of the diseased parts was proposed, and assented to. On the 4th day of August, 1826, it was performed, with the loss, certainly, of much blood, and the infliction of considerable pain: one vessel only, however, was tied, and the pain and the hæmorrhage ceased with the operation. At the end of the fourth week the wound was perfectly healed; the natural form of the parts was preserved; and I do not recollect to have observed a smoother or more perfect cicatrix. The cure has been complete.

The morbid growth in cases of this kind, of which in the course of thirty years I have seen three examples, is, I believe, altogether, simple and harmless, otherwise than as it incommodes by its bulk and pressure. It is quite destitute of malignant character, and appears to consist entirely of redundant growth of the common integuments of the parts. In the present instance, as in Mr. Hey's observations, its rate of increase in the earlier stages was extremely slow; latterly it grew rapidly; and at length, by its bulk, by its pressure upon contiguous parts, and by the deformity it occasioned, it became a very pressing evil.

Of the sketches, one was made two days before the operation, and the other seven weeks after it. They exhibit a very faithful view both of the morbid appearance, and of the result of the treatment.

The part removed, in one entire portion, is preserved in my collection, and forms, in its kind, a very perfect pathological specimen."

The above case I have given in the operator's own words. The following one occurred to me in the summer of 1831.

Mr. S., a gentleman, in the eighty-second year of his age, applied to me with a tumour of the character just described. He had had an enlargement of the nose for several years, which commenced with simple redness of the extremity; its first progress was exceedingly slow, but at length the cheeks participated not only in the redness, but also in the warty or tuberculated character of the nasal tumour. Within the last few years the disease has advanced with greater rapidity; but still, from the advanced age of the patient, and the inconvenience being at this time limited to the personal deformity, he was rather discouraged from thinking of the operation, and simply recommended to squeeze out the sebaceous matter from the orifices of the enlarged follicles, and occasionally to apply some simple emollient to prevent the chafing or excoriation of the skin. Mr. S. was a very fine old man, with all the liveliness and courage belonging to a much earlier period of life; and, having heard of the preceding case, in which my father had removed the diseased mass, he was not only willing but desirous to submit to a similar operation. He stated to me that his comforts were much circumscribed, in consequence of the unsightly appearance he presented; and that he was restrained from associating with his friends, entirely from delicacy of feeling towards the strangers he might accidentally meet in society. Whenever he went into public, (and from his occupations he was much abroad,) his endeavour to conceal the tumour had made him contract an ungainly stoop, which gave him the appearance of considerable decrepitude. Altogether, he suffered much from mental uneasiness, although in all other respects he was in the enjoyment of perfect, even of robust health.

I had several opportunities of watching the progress of this case, and at the end of eighteen months the tumour had increased rapidly in bulk, the respiration had become embarrassed, and the skin excoriated in two or three places. The following is the appearance it now presented, (May 1831.) The enlargement of the integuments commences almost immediately below the depression dividing the nose from the forehead, the skin gradually increasing in thickness as it approaches the extremity of the organ. At this point, from its weight and excessive development, it depends nearly an inch below the level of the lower lip. The left ala nasi is occupied by a nodule of a similar nature, about the size of a walnut, and is separated by a deep fissure from the larger portion, between which the skin is slightly excoriated: the right ala is also enlarged though not to the same extent. The nares are closed by the pressure of the tumour, and from the loss of elasticity in the cartilages; but at the same time the internal area of the nostrils, as well as the length of these openings, is much increased, apparently from the dragging or stretching produced by the weight of the diseased mass.

The sebaceous follicles are greatly enlarged; the whole tumour is of a deep red colour, with small varicose veins meandering over its surface: the skin of the cheeks is red, hard, and tuberculated.

The health of Mr. S. being still perfectly firm, the operation was determined on, under the sanction of the late Dr. Babington, in whose presence it was performed a few days after our consultation.

An incision was commenced at the outer or posterior convexity of the right ala nasi, carried up over the bridge of the nose, about an inch below the fronto-nasal depression, and terminated at the inner or anterior convexity of the left ala. The skin was now dissected off, and by repeated strokes of the scalpel detached from the subjacent cellular tissue; in fact the operation consisted in *peeling* off the integuments, leaving beneath a sufficient layer to prevent any danger of opening the chambers of the nose. Owing to the denseness of the parts, there was great difficulty in reflecting the diseased skin, and, obscured as the operation was by a pretty copious hemorrhage, the *modelling* of the new organ was a matter that required a little time and caution. The point of the finger of the left hand was inserted into each nostril, as the dissection advanced over these parts, in order to regulate by the touch the necessary thickness of skin that required to be left as the substratum of the future granulations. At about the end of four minutes the mass was detached, and the nodule upon the left ala nasi, which was more pendulous than the larger portion, was removed by a single stroke of the scalpel. Several small arteries bled actively from the exposed surface, but were soon closed by a little pressure. The wound was covered with a light dressing, and the patient kept his bed during the rest of the day; the loss of blood probably did not exceed eight ounces.

At the end of the fourth day the dressings were removed, and the general surface of the wound was found covered by a layer of soft lymph. After a few days, healthy granulations sprouted up, and the process of cicatrization advanced rapidly. At the end of about four weeks the sore was perfectly healed, without one untoward symptom having arisen during the process of cure. Mr. S. is now perfectly well, the tumour never having again made its appearance. Owing to the tuberculated state of the cheeks, and their red hue, the cicatrix, which gradually contracted more and more, is scarcely distinguishable from the contiguous skin.

The principal difference in the operation just described from that performed by Mr. Hey, consisted in commencing the incision on the outer or larger circumference of the tumour; and in dissecting, in consequence, from above downwards, the steps of the operation were less observed by the bleeding than if taken in a contrary direction. The skin at the upper part also, being less densely connected with the subjacent cellular tissue, affords a greater facility of reflecting the integument, which is of considerable consequence to the operator, especially as he approaches the extremity of the nose. One caution is yet to be added. When detaching the skin from the situation of the ala nasi, the fore finger of the left hand should constantly be kept in the nostril, since the great dilatation of these cavities makes it necessary to beware lest the chambers of the nose be exposed by the scalpel of the operator.

## Figures and Tables

**Figure f1:**
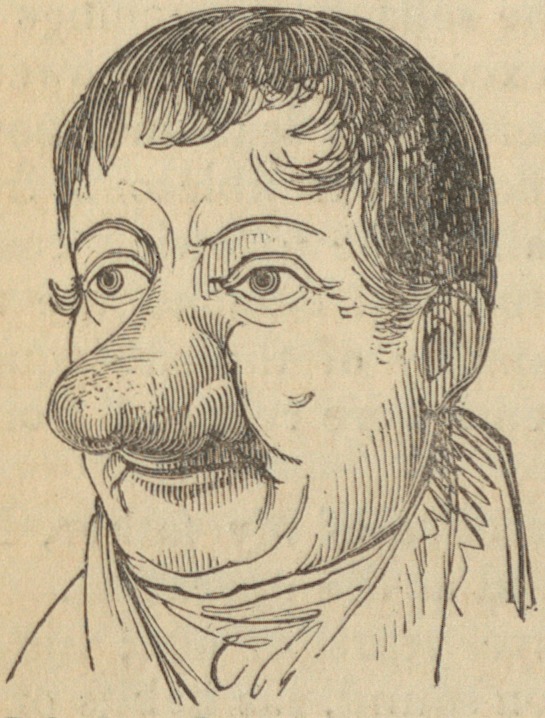


**Figure f2:**